# Determinants of smokeless tobacco use and prevalence among Sudanese adolescents

**DOI:** 10.1186/s13690-021-00699-w

**Published:** 2021-10-12

**Authors:** Mohammed Othman, Nik Daliana Nik Farid, Nasrin Aghamohammadi, Mahmoud Danaee

**Affiliations:** 1 Department of Social and Preventive Medicine, Faculty of Medicine, University of Malaya, Kuala Lumpur, Malaysia; 2 Centre for Epidemiology and Evidence-Based Practice, Department of social and Preventive Medicine, Faculty of Medicine, University of Malaya, 50603 Kuala Lumpur, Malaysia

**Keywords:** Adolescent, Smokeless tobacco, Sudan, Toombak, Urban health

## Abstract

**Background:**

Smokeless tobacco is a part of social and cultural life in Sudan. The affordability and availability of this kind of tobacco make it a fundamental issue in adolescents. The aim of this study is to investigate the extent of the use of smokeless tobacco in adolescents and its determinants.

**Methods:**

A school-based cross-sectional study was conducted in Khartoum state in Sudan. The study targeted male and female adolescents in secondary schools. A total of 3387 students from public and private schools participated in the study. Multistage random sampling was used to select the participants. The Arabic version questionnaire from the Global Youth Tobacco Survey (GYTS) was utilised to collect the data from the participants.

**Results:**

Among the participants, 57.3% were females and 42.7% were males. Students from private and public schools were 48.4 and 51.6%, respectively. The overall prevalence of those who had ever used smokeless tobacco was 7.6%, in which the prevalence among male students was 11.0% while among females was 5.0%. The determinant factors were male gender (OR 1.53 CI 95% 1.03–2.28), family structure (OR 1.52 CI 95% 1.03–2.23), exposure to second-hand smoke at home (OR 1.60 CI 95% 1.11–2.31), friends smoking cigarettes (OR 1.78 CI 95% 1.22–2.60), lack of restriction of selling tobacco to minors (OR 1.73 CI 95% 1.25–2.39), promotion of smokeless tobacco (OR 2.12 CI 95% 1.20–3.72) and low self-efficacy (OR 7.47 CI 95% 4.45–12.52).

**Conclusion:**

A comprehensive prevention programme that enforces the prohibition of the promotion of smokeless tobacco and the selling of smokeless tobacco to minors is crucial. Moreover, the prevention programme should enhance adolescents’ self-efficacy.

**Supplementary Information:**

The online version contains supplementary material available at 10.1186/s13690-021-00699-w.

## Background

Smokeless tobacco is any kind of tobacco that is consumed through mouth or nose without any burning. Chewing and dipping (sucking) are the most prominent forms of using oral smokeless tobacco [1]. Different types of smokeless tobaccos are available according to their plant species, nicotine concentration and additives.

The most prevalent type of smokeless tobacco in Sudan is toombak which was introduced approximately 400 years ago [2]. Toombak is planted, manufactured and produced locally. It is prepared from dried leaves of *Nicotiana rustica* that are mixed with natron (sodium bicarbonate) solution. The mixture is fermented for 24 h before use [1, 3]. *Nicotiana rustica* contains up to 18% *nicotine*, which is more than *Nicotiana tabacum* that consists 0.5 to 9% nicotine [4].

Toombak dipping is practiced by taking a small portion from the toombak bag, putting it in the palm of the other hand to manipulate until it becomes a round solid mass. This mass is called saffa and it is weighted approximately about 10 g [3]. Saffa is mainly placed in three sites within the mouth; either between the gum and lip or between the cheek and lip or on the floor of the mouth [3]. It is left to absorb slowly for up to 1 h or until it becomes bland; however, an average of a saffa dipping is 10–15 min [3]. Adolescent choose to make a small saffa and dip it between the upper lip and gum, thus, it is unnoticeable and easily hidden [3].

Natron is added to toombak to homogenise the powder to have an alkaline effect which increases the pH to a range of 11.0–11.8. A high level of pH in tobacco products increases the absorption of nicotine; hence it increases its activity. Nicotine concentration in toombak, used in Sudan is 32.2–102.4 mg/g, which is two times higher than smokeless tobacco (snuff) used in the USA and five times higher than the concentration of nicotine used in smokeless tobacco (snus) in Sweden [3]. Thus, toombak is highly addictive and cannot be resisted [3].

Additionally, many types of tobacco N-nitrosamines such as N-nitrosonornicotine (NNN), 4-(methylnitrosamino)-1-(3-pyridyl)-1-butanone (NNK),N-nitrosoanatabine (NAT) and N0-nitrosoanabasine (NAB) are quantified in high levels in toombak products and the saliva of toombak users. The level of N-nitrosamines in toombak is 100 folds higher than thst found in Swedish snus, and this can be attributed partially to the use of *Nicotiana rustica* plants, the fermentation process of toombak in high temperatures, prolonged storage and contamination during the process [2, 5].

Toombak usage was associated with oral cancer especially at the site where saffa was placed, and this was a result of a high level of N-nitrosamines in toombak. In Sudan, it was documented that the odds ratio of lip cancer in toombak users was OR 7.3(4.3–12.4) and the odds ratio of buccal and mouth floor cancer was OR 3.9 (2.9–5.3) [3]. Even more, the odds ratio of lip cancer increased in the long term for toombak users to reach OR 73.0 (9.8–542.2) [3]. This means that it has a dose-response relationship [3]. Furthermore, the use of toombak was associated with tumours of the salivary glands [6].

The prevalence of those who ever used toombak among 14–17-year-old Sudanese was 1.7%. However, this percentage increased sharply to 11.3% in 18–19 year-olds [2]. In 2010, among Sudanese secondary school students, the percentage of those who had tried toombak was 8.1% and that for the current users was 3.5%. Whereas, in 2014 GYT survey, the prevalence increased to reach 13.2% for those who had tried toombak and the percentage of current users was 4.9% [7]. However, in 2017, the prevalence of those who had tried toombak decreased to 10.9% [8]. In Sweden, smokeless tobacco is called snus and it was reported that the percentage of those who ever used snus among Swedish students in the 9th grade was 54.6% for males and 32.3% for females [9].While the prevalence of smokeless tobacco and betel quid in Pakistani secondary school students was 42.6% [10]. In India, the chewing tobacco (Gutka) percentage was 10.8% among the sixth and eighth grade students in Delhi and Chennai [11].

A few studies in Sudan have addressed the issue of smokeless tobacco use in adolescents. Therefore, there is insufficient information on its use and its determinants in Sudan. None of the studies in Sudan have linked the family structure, allowance, grade, or academic performance with smokeless tobacco use and only one study has addressed the usage of smokeless tobacco in public and private schools.

Developed countries have advanced surveillance system that can spot and monitor tobacco use in adolescence. However, most of the developing countries do not have a system in place to monitor thus, tobacco-related problems may be underestimated [12]. In Sudan, one-fifth of the population is adolescents who are under the age of 19 [13]. Therefore, health concerns and any matters that affect adolescents’ health should be investigated and addressed. However, tobacco use in adolescence is seen as a less priority. Meanwhile tobacco is a major cause of preventable deaths as many users start consuming tobacco in early adolescence, which increases their risk because of the longer duration of tobacco use [14]. It is worth mentioning that there is no official tobacco control policy specifically targeting adolescents in Sudan [15]. The burden of tobacco use, morbidity and mortality will increase unless effective control and prevention programmes are developed and implemented in the near future [16]. These prevention programmes should target adolescents who are at high risk of tobacco use. The current study aims to identify the determinant factors of smokeless tobacco use and also to estimate the prevalence among adolescents.

## Methods

The state of Khartoum is divided into three cities which includes seven localities according to administrative divisions. All seven localities were considered as strata and were included in the study. Moreover, schools in the localities were divided into strata according to the type of schools (public and private) and gender (boys and girls). This division produced four strata of schools; 216 public boys schools, 206 public girls schools, 264 private boys schools and 357 private girls schools. Consequently, from each stratum in each locality, one school was selected through a simple random sampling. As a result, 28 schools were selected for the study. However, a girls school in Omdurman locality was found to have an insufficient number of students, hence, another girls school from the same locality was added to fulfil the number needed for the sample size. Thus, a total of 29 schools participated in the study. At the school level, classes were divided into three strata according to grades (1st grade, 2nd grade, 3rd grade). In every participating school, a class was selected by simple random sampling from each grade. Through universal sampling, all students in the selected classes were targeted to participate in the study. Supplementary file 1.

The sample size of this study was calculated with open source epidemiological statistics for public health version 3.01 OpenEpi, Population size 205,319, anticipated frequency (p) 13.1% [17], absolute precision (d) 3%, design effect (deff) 3. According to OpenEpi, 1455 participants were needed. However, the state has a large, diverse population, thus it is a heterogeneous community. Therefore, the number of participants needed was doubled. Moreover, the sample size was increased by 15% to overcome nonresponse. Consequently, the number of participants needed for the study was set to 3304 students.

After sampling, the number of participants from each locality was ensured to be in proportion to the size of the locality. Hence, localities with larger population had more students in the study than the smaller ones. Eventually, 3387 students participated in the study.

### Outcome and determinants

The primary outcome ‘has ever used toombak’ was defined as the participant having used toombak, even if it was one dip, at any time prior to the study meanwhile if a participant had used toombak in the past 30 days, they were considered as ‘current toombak user’. Data on the following socio-demographic characteristics were obtained from participants: age, gender, grade, academic performance, daily allowance (pocket money), type of school, location of the school, locality, family structure, and parents’ education level. The determinants of toombak use consisted of exposure to second-hand smoke, pro and anti-tobacco messages, parents smoking cigarette, friends smoking cigarettes and using smokeless tobacco, teachers smoking cigarettes and using smokeless tobacco, self-efficacy and restriction of selling toombak to minors. As for the outcome for ever used toombak, the question was: Have you ever tried or experimented using toombak, even once? Yes/No.

### Data collection

The study was granted ethical approval from the Medical Ethical Committee at the University Malaya Medical Center in September 2015 (MECID.NO: 20159–1666). In September 2016, the study received approval from the National Research Ethics Review Committee in the Federal Ministry of Health, Sudan. Subsequently, approval from the Ministry of Education in Khartoum State was obtained to carry out the study in the schools in the state. The aim and objectives of the study were explained to the schools’ principals. Written consents for participating students were obtained from parents and students, after explaining the objectives of the study and ensuring confidentiality to the students.

Within 2 months, from September to October 2016, standard anonymous self-administered questionnaires were distributed to collect data from the students in the classroom by a member of the survey team without the presence of school personnel in the classroom. A validated Arabic version of the Global Youth Tobacco Survey questionnaire was adopted to be utilised in the study [18]. The Global Youth Tobacco Survey (GYTS) is an anonymous, self-administered, standardized, school-based survey of students designed to collects data on tobacco use among young people to monitor and guide the implementation and evaluation of tobacco prevention and control programmes [18]. The GYTS questionnaire included all the objectives of the study. Furthermore, it has been used in many previous studies in Sudan; hence, it will facilitate the comparison. The questionnaire was consisted of 76 questions, all of which were close-ended except for two. The questionnaire was designed to prevent students from skipping questions. The completion of the questionnaire by the participants took approximately 30 to 40 min. The questionnaire was pre-tested in 19 students and adjusted accordingly. Those who participated in the pre-test were excluded from the main study.

### Data analysis

IBM Statistics Package for the Social Sciences (SPSS) version 20 was used to analyse the data. Due to the use of multistage sampling method in the sampling process, a complex sample analysis was performed in analysis. Moreover, the weight of each participant was calculated and utilized accordingly. At first, the schools weight was calculated proportionally to the number of schools in the locality and afterword, the class weight was determined in ratio to the number of classes in the school. Descriptive statistics of the sociodemographic characteristics of the participants were given in terms of frequencies and percentages. To elicit the determinants that may contribute to toombak usage, a model was developed. Using univariate analysis, sociodemographic characteristics and other associated factors were obtained through complex samples General Linear Model. *P*-value was set to 0.25 because using *p*-value of 0.05 is very strict and might not identify important variables [19]. All variables above 0.25 were excluded. The univariate analysis for sociodemographic characteristics and for other associated factors was done for each group separately. Subsequently, significant sociodemographic characteristics and other associated factors from the above univariate analysis with *p*-value less than 0.25 were selected to undertake complex samples multivariate logistic regression for each group separately and at this step the *p*-value was set to 0.05. The crude model was obtained from this step. Following that, the significant sociodemographic characteristics and other associated factors form the previous logistic regression was combined together to process under multivariate logistic regression. Step-by-step backward selection was used in multivariate logistic regression. Consequently, this produces the adjusted model for the study. The result was expressed in odds ratio (OR) with a 95% confidence interval (CI) and the *p*-value was considered significant if it was less than 0.05.

## Results

The response rate of the study was 100% for schools and students. Female students accounted for 57.3% of students while males accounted for 42.7%. Students from public schools were 51.6% whereas 48.4% were from private schools. The overall of students who participated in the study were 3387 (Table 1). Overall, 78.2% of the students were living with both parents and only 1.1% of the students were enrolled in rural schools. Mean daily allowance was 8.8 Sudanese pounds, 95% CI (8.57–9.04) and the standard error was 0.119.

**Table 1:** Sociodemographic characteristics of participants

Characteristics	Unweighted Counts	Weighted Percentage %	95% CI
**Locality**			
Khartoum	422	17.9%	16.4–19.5
Jabal Awliya	518	14.0%	12.9–15.2
Omdurman	459	12.5%	11.5–13.6
Karari	442	11.2%	10.2–12.2
Ombada	647	17.2%	16.0–18.4
Bahri	375	10.1%	9.2–11.1
East Nile	511	17.2%	15.9–18.6
Missing	–		
**Location of school**			
Urban	3266	98.9%	98.5–99.2
Rural	108	1.1%	0.9–1.3
Missing	–		
**Type of School**			
Public	2035	51.6%	50.0–53.2
Private	1339	48.4%	46.6–50.3
Missing	–		
**Age**			
14 years or less	726	21.8%	20.4–23.2
15 years	879	24.8%	23.4–26.3
16 years	964	28.6%	27.1–30.2
17 years above	783	24.8%	23.3–26.3
Missing	22		
**Daily Allowance**			
Low ≤5	1240	35.7%	34.1–37.3
Average 6–10	1461	48.8%	47.0–50.6
Above Average 11–15	245	9.7%	8.6–10.9
High 16–20	107	4.7%	3.9–5.6
Very High 21–50	19	1.0%	0.6–1.6
Missing	302		
**Gender**			
Male	1604	42.7%	41.1–44.3
Female	1770	57.3%	55.5–59.0
Missing	–		
**Grade**			
1st secondary	1098	31.2%	29.9–33.0
2nd secondary	953	23.5%	22.2–24.8
3rd secondary	1323	45.3%	43.5–47.1
Missing	–		
**Academic Performance**			
Excellent	615	20.3%	18.9–21.8
Very good	1430	44.1%	42.4–45.8
Good	1064	29.7%	28.2–31.2
Fair	160	4.6%	4.0–5.3
Poor	42	1.3%	1.0–1.8
Missing	63		
**Family structure**			
Father only	74	1.8%	1.4–2.3
Mother Only	374	11.0%	10.0–12.1
Father and Mother	2616	78.2%	76.8–79.6
Other	294	9.0%	8.1–10.0
Missing	16		
**Father’s level of education**			
Illiterate	140	3.7%	3.1–4.4
Can read and write	470	12.7%	11.7–13.8
Primary	210	5.8%	5.1–6.6
Elementary	296	8.6%	7.7–9.6
Secondary	538	15.2%	14.1–16.4
University	665	21.3%	19.9–22.8
Higher Education	333	12.4%	11.2–13.7
Do not know	694	20.4%	19.1–21.8
Missing	28		
**Mother’s level of education**			
Illiterate	290	8.0%	7.2–8.9
Can read and write	375	10.4%	9.4–11.4
Primary	334	9.4%	8.5–10.5
Elementary	392	11.6%	10.6–12.7
Secondary	666	19.5%	18.2–20.9
University	573	19.4%	18.0–20.9
Higher Education	173	5.9%	5.1–6.8
Do not know	543	15.9%	14.7–17.2
Missing	28		

The overall prevalence of those who had ever used smokeless tobacco (toombak) was 7.6%. Among male students, the prevalence was 11.0% while it was 5.0% in female students.

Only city, gender and family structure were the associated sociodemographic factors with those how had ever used smokeless tobacco. Concerning other determinant factors; exposure to second-hand smoke at home, friends smoking, receiving free smokeless tobacco, low self-efficacy and lack of restriction of selling tobacco to minors were associated with smokeless tobacco use in students. Living in Bahri city was a determinant of ever use of smokeless tobacco with (OR 1.58 CI 95% 1.11–2.25) (Table 2). Male gender was also a significant determinant (OR 1.53 95% CI 1.03–2.28). Living with one parent or others was a determinant (OR 1.52 95% CI 1.03–2.23). Being in the third grade was a determinant for smokeless tobacco use; however, after adjustment, it turned out to be insignificant. Moreover, exposure to second-hand smoke at home was associated with smokeless tobacco use (OR 1.60 95% CI 1.11–2.31). Friends smoking cigarettes was also a significant determinant for smokeless tobacco use (OR 1.78 95% CI 1.22–2.60). A strong determinant was low self-efficacy (OR 7.47 95% CI 4.45–12.52). Receiving free smokeless tobacco was also associated with smokeless tobacco use in students (OR 2.12 95% CI 1.20–3.72). Lack of restriction of selling smokeless tobacco to minors was another factor associated with ever using smokeless tobacco with (OR 1.73 95% CI 1.25–2.39).

**Table 2:** Crude and adjusted models for Independent variables for ever used toombak

Variable	Total n	Ever used toombak	Never used toombak	Crude OR (univariate)		Adjusted OR (multivariate)	
				OR CI 95%	***P***-value	OR CI 95%	***P***-value
**City**							
Khartoum	926	63 (2)	863 (29.9)	1.08 (0.73–1.61)	< 0.003	1.08 (0.70–1.67)	< 0.034
Omdurman	1526	111 (3)	1415 (37.8)	Ref		Ref	
Bahri	873	98 (2.5)	775 (24.7)	1.82 (1.27–2.63)		1.58 (1.11–2.25)	
**Gender**							
Male	1576	189 (4.7)	1387 (37.9)	2.30 (1.65–3.22)	< 0.001	1.53 (1.03–2.28)	< 0.032
Female	1749	83 (2.9)	1666 (54.5)	Ref		Ref	
**Grade**							
Ist	1081	64 (1.6)	1017 (29.6)	Ref	< 0.024	Ref	< 0.232
2nd	942	75 (1.9)	867 (21.6)	1.49 (0.99–2.24)		1.29 (0.83–2.00)	
3rd	1302	133 (4.1)	1169 (41.3)	1.66 (1.14–2.41)		1.40 (0.94–2.08)	
**Family structure**							
Father and mother	2576	198 (5.3)	2378 (72.9)	Ref	< 0.010	Ref	< 0.032
Others	733	74 (2.3)	659 (19.5)	1.60 (1.11–2.30)		1.52 (1.03–2.23)	
**Exposure to second-hand smoke at home**							
Yes	807	106 (2.9)	701 (21.2)	1.60 (1.06–2.42)	< 0.024	1.60 (1.11–2.31)	< 0.010
No	2506	166 (4.7)	2340 (71.3)	Ref		Ref	
**Seeing someone smoke in school**							
Yes	673	101 (2.7)	572 (17.9)	1.46 (1.01–2.11)	< 0.041	1.40 (0.97–2.02)	< 0.065
No	2632	170 (4.9)	2462 (74.5)	Ref		Ref	
**Friends smoking cigarette**							
Yes	977	154 (4.3)	823 (24.7)	1.80 (1.12–2.89)	< 0.014	1.78 (1.22–2.60)	< 0.003
No	2334	117 (3.3)	2217 (67.7)	Ref		Ref	
**Restriction of selling toombak to minors**							
Yes	93	14 (0.4)	79 (2.1)	1.12 (0.34–3.73)	< 0.015	1.20 (0.39–3.71)	< 0.004
No	903	107 (2.9)	796 (23.3)	1.63 (1.17–2.28)		1.73 (1.25–2.39)	
I did not try to buy	2240	140 (4.2)	2100 (67.1)	Ref		Ref	
**Received free toombak from companies**							
Yes	179	45 (1.2)	134 (3.5)	2.26 (1.26–4.07)	< 0.006	2.12 (1.20–3.72)	< 0.009
No	3056	217 (6.3)	2839 (89)	Ref		Ref	
**Low self-efficacy** (Peer pressure for using toombak)							
Yes	116	63 (1.5)	53 (1.6)	7.32 (4.25–12.62)	< 0.001	7.47 (4.45–12.52)	< 0.001
No	3145	200 (6)	2945 (90.9)	Ref		Ref	

## Discussion

El-Amin study found that the percentage for ever used toombak was 8.1% in 2005 [15]. This increased in 2014 GYTS to reach 13.2% [7]. However, it declined in Almahdi study to reach 10.9% [8]. Moreover, it further dropped in this study to reach 7.6%. The current study had almost 99% of students from urban schools. Therefore, this might affect the prevalence of ever using toombak in this study since the use of smokeless tobacco is more prevalent among rural students [20]. The percentage of students who had ever used smokeless tobacco accounted for 11.2% in Egypt [21], 9% in Kenya, 12.5% in India, 16.2% in Nepal and 11.9% in Norway [8]. It seems that Sudan had the lowest rates among all the countries which have similar smokeless tobacco products as Sudan.

According to this study, males were more likely to have tried smokeless tobacco and it was statistically significant. Another study in Sudan demonstrated the same result when it reported that ever and current usage of smokeless tobacco were associated with the male gender among school students [15]. Moreover, in Saudi Arabia, a study found that users of smokeless tobacco were only males [22]. Despite the affordability of toombak and the ability to use it secretively without a trace of smoking smell in clothes, females usually avoid the use of smokeless tobacco in the Middle East region because it is labelled by communities as a masculine feature. A similar significant gender difference was also reported in a study in Swedish adolescents [9]. However, in a South African study, there was no gender difference in the current users of smokeless tobacco [20] because smokeless tobacco in South Africa was considered as women’s choice of tobacco [23].

The present study showed that not living with both parents was associated with ever toombak use. This was compatible with a study among ninth grade students in USA when it reported that ever smokeless tobacco use was associated with living with one parent [24]. The difficulties to provide continuous supervision and discipline [25, 26] in the absence of one or both parents in the family may have contributed to tobacco use [27]. It seems that experimenting with tobacco use was associated with not living with both the father and the mother, however, the decision to continue using tobacco is determined by other factors [24].

In this study, the grade level had no significant relation to ever smokeless tobacco use and moreover, there was no age difference in those had ever used smokeless tobacco in this study. A similar result also was noticed in a study in Congo which found that there was no significant association between current smokeless tobacco use and age [28]. The age range in secondary schools is too narrow to detect any difference. The differences might be apparent if comparison was made between students in secondary stage of schooling to primary stage of schooling [29]. Hence, the result of lack of association between grade, age and toombak use might be due to the narrow age range in the study, 14–18-year-olds. Nonetheless, the finding of the study might be authentic. Additionally, the present study showed no significant difference between urban and rural school students regarding their use of smokeless tobacco. The lack of association in the study can be due to under-representation of rural students. The finding of the study did not show any significant difference between public and private schools in those who had ever used smokeless tobacco. This might be clarified by the closeness of tobacco regulations and the similitude in students in public and private schools [29].

Many factors influence the academic performance of students besides tobacco use such as, interest in school and classroom environment, parental support, past achievement, academic motivation [30]. On the other hand, multiple factors determine tobacco use in students other than academic performance. Therefore, this may explain the lack of association between academic performance and smokeless tobacco use in the present study.

This study showed no significant association of daily allowance with having ever used smokeless tobacco. In Sudan, smokeless tobacco is planted, manufactured and produced locally, thus it is cheap and affordable. The cheap price of toombak, low taxation of tobacco products and the sharing nature of smokeless tobacco use in society might diminish the association between smokeless tobacco use and daily allowance [29].

Our study showed no significant association between parents’ level of education with having ever used smokeless tobacco. This finding was in agreement with another study in Sudan which showed the lack of association between parents’ level of education and ever using smokeless tobacco among secondary school students [8]. Tobacco use in adolescent is unacceptable in Sudanese community and parents oppose this behaviour irrespective of their level of education.

Exposure to second-hand smoke increases the sensitivity to nicotine and leads to the desire for nicotine use. Moreover, exposure to second-hand smoke in adolescents normalises the behaviour of using tobacco and puts a social pressure on adolescents to use tobacco to conform with their surroundings [31, 32]. These may explain the association between second-hand smoke at home and ever using smokeless tobacco.

Selling tobacco to minors was associated with having tried smokeless tobacco in the current study. Although selling tobacco to minors is prohibited in Sudan, many adolescents succeed in buying tobacco with the excuse that it is bought for an adult person. It is prevalent to send minors to buy tobacco for adults in Africa [33]. Sending minors to buy tobacco for adults was a risk factor for initiation of tobacco use in Nigeria and Gambia [33, 34].

The promotion of smokeless tobacco was associated with ever using smokeless tobacco among adolescents in the present study. This result was compatible with a finding from Congo-Brazzaville; when a study reported that pro-tobacco advertising was associated with the current use of smokeless tobacco in students, aged 12–17 years [28]. A systematic review of nine longitudinal studies from four countries conducted in 2003, concluded that exposure to tobacco advertising and promotion increases the likelihood of tobacco use in adolescents [35]. However, a study in Sudan showed that there was no significant association between tobacco advertising and intention to use smokeless tobacco [8].

Anti-tobacco advertising may counter the effect of pro-tobacco advertising but the outcome has not yet reached to the extent of decreasing the prevalence of tobacco use in adolescents [36]. Furthermore, not all anti-tobacco advertising campaigns have produced the desired effectiveness and impact. The ineffective anti-tobacco advertisement may have no effect on adolescents’ tobacco prevention and even more, it may produce an unintended effect. This may interpret the lack of association between anti-tobacco messages and ever using smokeless tobacco in this study.

The inability to reject smokeless tobacco when it was offered by a friend was associated with having tried smokeless tobacco in adolescents in the present study. This was compatible with a Bangladeshi study among rural secondary school students which established the association between low self-efficacy and smokeless tobacco use [37]. Low self-efficacy among adolescents may exhibit a favourable social and emotion perception about tobacco use [38].

El-Amin study in Sudan as well as this study have documented the lack of association between parents smoking and the use of smokeless tobacco in students [15]. The type of tobacco used by parents has an effect on the tobacco product used by adolescents. This was evident in studies from Sudan and Sweden [15, 39]. Both studies have shown no significant association between parents smoking cigarettes and smokeless tobacco use in adolescents; however, the smokeless tobacco use in parents was associated with current use of smokeless tobacco in adolescents [15, 39]. Moreover, El-Amin study reported that cigarette smoking among friends was positively associated with ever using smokeless tobacco among adolescents in Sudan [15]. The same result was documented in the current study. Adolescents might imitate the behaviour of their peers; however, in other cases, they might choose friends who had the same behaviour [40].

In this study, tobacco use in teachers was not associated with adolescents having tried smokeless tobacco. Older adolescents might not consider teachers as role models; therefore, they might not copy and imitate the behaviour of teachers. On the other hand, peers and friends might be more influential [41].

### Strengths and limitations

The novelty of current study is that it addresses the issues of smokeless tobacco in large sample size with high participation rate. Moreover; it addresses its association with family structure, allowance, grade, academic performance, location of school and type of school. In addition to exposure to second hand-smoke, selling tobacco to minors, low self-efficacy, pro and anti-tobacco messages, friends using tobacco, parents using tobacco and teacher using tobacco. The results of study can extrapolate through the state and moreover to the Sudan. On the other hand, the study targeted students only; therefore, it cannot be generalised to school drop-outs. Furthermore, the under representation of rural school students might obscure their characteristics and determinants; therefore, further study among these students is recommended. Moreover, addressing the issues of smokeless tobacco use in parents and tobacco use in siblings might have added more detailed information which could not be obtained from this study. At last, this is a cross-sectional study; thus, no causal relationship can be obtained from this study.

## Conclusion

The study can help spot high risk groups of adolescents through the identification of the determinant factors of using smokeless tobacco. Moreover, it can help design and develop prevention programmes that counter the determinant factors. Low self-efficacy is the greatest determinant of smokeless tobacco use in adolescents which increases smokeless tobaccos usage by seven folds; therefore, the prevention programmes should target and enhance self-efficacy of adolescents. The effect of the promotion of smokeless tobacco and selling tobacco to minors should be taken into consideration since it points to the lack of enforcement, efficacy of prohibition of tobacco promotion and restriction of selling tobacco to minors. Augmentation and reinforcement of legislations that prohibit promotion of tobacco and restrict selling tobacco to minors is fundamental in tobacco prevention. Further research is needed to investigate on rural students and drop-outs from schools in addition to monitoring the trend of smokeless tobacco use.

## Supplementary Information

**Additional file 1.**

## Data Availability

The data that support the findings of this study are available from Ministries of Health and Education in Sudan. However, restrictions apply to the availability of these data, which were used under license for the current study, and so are not publicly available. Data are however available from the authors upon reasonable request and with permission of Ministries of Health and Education in Sudan.

## Abbreviations

CI: Confidence interval
GYTS: Global Youth Tobacco Survey
OR: Odd ratio
