# Supplementary figures and images for: Determinants of smokeless tobacco use and prevalence among Sudanese adolescents
Source: Arch Public Health. 2021 Oct 12;79:176. doi: 10.1186/s13690-021-00699-w (PMC8507347; doi:10.1186/s13690-021-00699-w)

**Supplementary file 1: Sampling procedure**


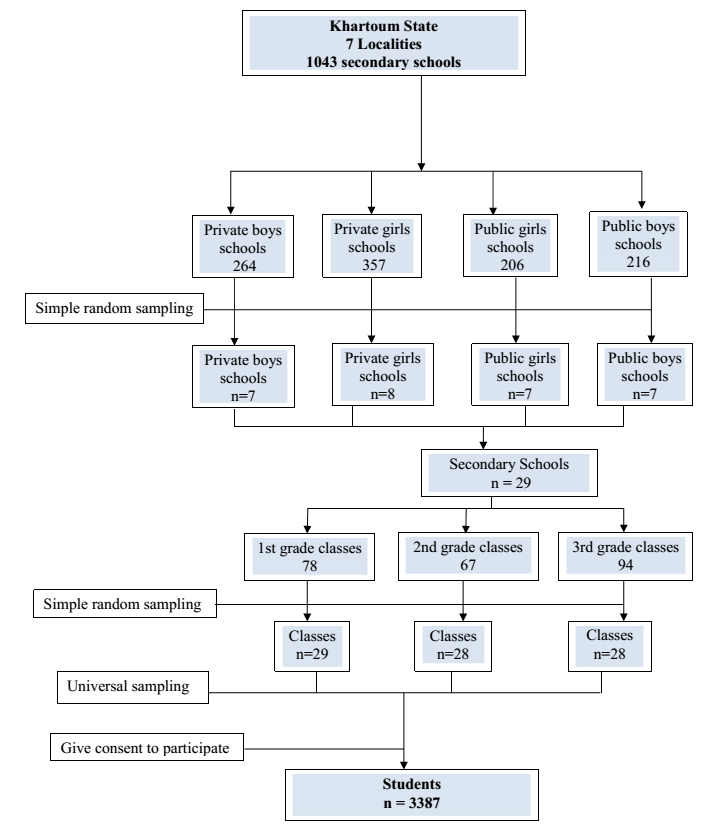

Supplement: Supplementary file 1 — Additional file 1. [file 13690_2021_699_MOESM1_ESM.docx]
